# Comparative evaluation of 4DCT and 4DCBCT for motion and volume measurement accuracy in a dynamic phantom

**DOI:** 10.1002/acm2.70489

**Published:** 2026-02-24

**Authors:** Bhumika Handa, Satyapal Rathee

**Affiliations:** ^1^ Medical Physics Division Department of Oncology University of Alberta Edmonton Alberta Canada

**Keywords:** 4DCBCT, 4DCT, SBRT, Motion management

## Abstract

**Background:**

Effective motion management is essential for accurate treatment planning, target localization and dose delivery in lung stereotactic body radiotherapy (SBRT). Lung tumors typically move 5–30 mm in superior‐inferior (SI) direction. The internal target volume (ITV) may be expanded up to 5 mm for day‐to‐day setup variations. Four‐dimensional cone beam computed tomography (4DCBCT), with a rotation scan time of 120 seconds, has the potential to improve on‐line target localization; however, its accuracy in target motion and volume measurements should be compared to four‐dimensional computed tomography (4DCT) with a scan time of < 60 seconds.

**Purpose:**

To compare the accuracy of 4DCT and 4DCBCT in measuring target motion and volume in a phantom programmed with both regular and patient‐derived irregular respiratory waveforms.

**Methods:**

A thorax phantom with a known target was imaged using 4DCT and 4DCBCT under controlled sinusoidal and irregular breathing patterns. 4DCBCT images were reconstructed using both Basic (i.e. FDK) and Advance (i.e. modified Mckinnon‐Bates) algorithms where the Advance algorithm subtracts re‐projection of *prior* image (using the entire un‐binned projections) from phase sorted projections to reduce streak artifacts. Motion amplitudes were calculated from target centroids across respiratory phases, as well as from 3DCBCT‐based motion blur profiles. Target volumes were assessed in average intensity projection (AIP) and maximum intensity projection (MIP), and in the reconstructed phases. Measured amplitudes were compared against programmed values, while the target volumes were compared in AIP, MIP and the phases between 4DCT and 4DCBCT.

**Results:**

For regular motion, 4DCT demonstrated the smallest deviations from the programmed amplitudes (SI, Left‐right (LR) and Anterior‐posterior (AP) 95% confidence intervals (CI): −0.8 to 0.1 mm, −0.3 to 0.2 mm and −0.5 to 1.1 mm respectively). In regular motion, 4DCBCT Advance algorithm slightly underestimated the SI motion by 1 mm (SI CI: −1.5 to −0.4 mm, LR CI −1.4 to 1.2 mm, AP CI −1.2 to 0.6 mm) while the Basic algorithm provided slightly larger variability (SI CI: −1.1 to 0.7 mm, LR CI −1.1 to 0.6 mm, AP CI −0.9 to 1.3 mm). 3DCBCT‐based estimates showed higher variability across all motion directions (SI CI −1.7 to 1.6 mm, LR CI −1.9 to 1.6 mm, AP CI −1.6 to 1.0 mm). For irregular motion, both 4DCT and 4DCBCT Advance yielded differences from average programmed amplitude with no consistent superiority. The measured target volume showed strong agreement between 4DCT and 4DCBCT. The target volume expansion due to within‐phase blurring showed a very similar trend between 4DCT and 4DCBCT Advance.

**Conclusions:**

4DCBCT within clinical workflow provides comparable motion and volume measurement accuracy to 4DCT. 3DCBCT estimated motion amplitude showed large differences from the programmed amplitude. 4DCBCT offers explicit and confident motion resolution, and phase‐dependent target volume at the time of treatment. These findings support the feasibility of integration of 4DCBCT, pending clinical validation, for image guidance in lung SBRT, especially where motion consistency between simulation and treatment is uncertain. Patient studies are needed to capture the influence of patient‐specific breathing irregularities such as hysteresis, baseline drift or anatomical deformation.

## 1 INTRODUCTION

Lung cancer remains the leading cause of cancer‐related mortality globally, with non‐small cell lung cancer (NSCLC) accounting for nearly 85% of all cases.^1^ Treatment options for a localized tumor typically include surgery or radiotherapy; the choice depends on tumor characteristics and patient condition.^2^ In many cases, radiotherapy is a key component of treatment, particularly when surgery is contra‐indicated. However, treating lung tumors with radiotherapy poses significant challenges. These include tumor motion due to breathing (may range from 5–30 mm in superior‐inferior (SI) direction), proximity to critical structures (e.g., spinal cord, esophagus), and the general thoracic anatomy. Radiotherapy's role has been continuously redefined through technological advancements, particularly in imaging, motion management, and delivery precision.^2^, ^3^ Advancements in radiation delivery techniques have improved treatment precision and outcomes by sparing healthy tissue and reducing side effects.^3^ Stereotactic body radiation therapy (SBRT) represents a paradigm shift in lung cancer radiotherapy, allowing high dose, hypo‐fractionated treatment delivery with good geometric accuracy.^3^, ^4^ With margins reduced and normal tissue sparing maximized, SBRT has achieved excellent local control in the early‐stage NSCLC, in medically inoperable patients.^1^, ^2^ Precise tumor delineation is critical in SBRT, and tumor motion due to respiration complicates this task. Therefore, motion management and advanced imaging techniques, such as four‐dimensional computed tomography (4DCT), are essential for treatment planning to satisfy the dose constraints for organs at risk.^3^ Effective motion management is essential for accurate on‐line target localization and dose delivery. Strategies range from motion‐encompassing approach, which creates the internal target volume (ITV) to include tumor motion, to more sophisticated techniques like active breathing control, voluntary breath‐old, and respiratory gating.^5^ The choice of technique depends on factors such as tumor size, location, patient compliance, and equipment availability. In general, an additional expansion of ITV by 3–5 mm is added to account for day‐to‐day patient setup and breathing uncertainties.

Both 4DCT and four‐dimensional cone beam computed tomography (4DCBCT) produce 3D image sets corresponding to each breathing phase (typically 10). However, there are major differences between the two in terms of data acquisition and image reconstruction. 4DCT is implemented on a CT scanner using low‐pitch helical^6^ with projection space binning, cine‐axial^7^ or intelligent cine‐axial^8^, ^9^, ^10^ with image space binning approaches. Radiotherapy 4DCBCT is implemented on a c‐arm medical linear accelerator^11^ with projection space binning. In a low‐pitch helical 4DCT approach used in this investigation, the couch speed is reduced (i.e. pitch is lowered) such that each image voxel remains illuminated by the beam during the whole breathing period. For regular breathing, this condition is sufficient to minimize binning artifacts.^12^ Each measured projection is tagged with projection angle, couch position, and breathing phase. This projection data is interpolated in couch motion and breathing phase directions to obtain sufficient data to reconstruction images for each desired breathing phase and couch position. In cine‐axial 4DCT, images are continuously obtained and phase tagged at a stationary couch position for fixed duration (generally an average breathing period plus a typical margin of 1 second). The couch is moved to the next position and the process is repeated.^7^ If a particular breath is longer than the fixed duration, the data for certain phases may be missed. The intelligent cine‐axial 4DCT adapts the scan duration at each couch position based on the current breathing period to minimize this problem.^8^, ^9^, ^10^ In the 4DCBCT approach, the acquired projections are binned into respiratory phases prior to image reconstruction and the linac gantry is slowed to increase the number of projections in each breathing phase.^11^ Therefore, it is important to compare the 4DCT and 4DCBCT imaging methods in phantom and patient studies. Several studies have made this comparison for one integration of 4DCBCT with a treatment unit, i.e. Elekta XVI system^13^, ^14^ in phantom. Furthermore, the effect of varying gantry speed on image quality and motion measurement accuracy has been studied for this system.^15^, ^16^, ^17^

Patient studies have highlighted the clinical advantages of integrating 4DCBCT for image guidance in SBRT of early‐stage lung cancer.^18^, ^19^, ^20^ Using the registration between end‐exhale phases of 4DCBCT and 4DCT as the reference,^18^ target localization with automatic soft‐tissue registration of the composite 4DCBCT image to ITV was better than the similar registration of 3DCBCT to ITV (3D vector error: 2.25 ± 0.44 mm vs. 3.59 ± 0.48 mm, *p* < 0.001) in 20 stage 1A non‐small cell lung cancer (NSCLC) patients. Similarly, manually corrected registration between the 3DCBCT blurred target and the ITV contour was worse by 1.9 ± 0.9 mm than 4DCBCT based automatic registration in 21 lung SBRT patients.^19^ Registration between the average intensity projections (AIP) from 4DCBCT and 4DCT was better than between 3DCBCT and AIP from 4DCBCT for reducing localization error (0.69 vs. 0.99 mm, *p* < 0.05)^20^ when the motion amplitude reduced by 30% between 4DCT and 4DCBCT acquisitions. All these studies have been conducted using the 4DCBCT on Elekta XVI.

Since all previous studies evaluated the Elekta XVI system, for the Varian's integration of 4DCBCT with linac, comparison of 4DCT and 4DCBCT using phantoms has not been made. The vendor specific differences in scan time and projection count may result in differences in streak artifacts. Additionally, Varian's system provides two reconstruction algorithms. The “Basic” algorithm reconstructs the binned projections using the FDK algorithm.^21^ Since the number of projections per phase bin is too small in 4DCBCT, despite slow acquisition, the binned phase images suffer from streaking artifacts due to view aliasing.^22^ Therefore, the system provides an “Advance” algorithm^11^ as a modification^23^ to Mckinnon‐Bates algorithm^24^ to further reduce the streaking artifacts. Advanced algorithm modifies the average image reconstructed from all the projections by adding the image of moving anatomy for each phase to reduce the streaking artifacts.

To our knowledge, this is the first quantitative phantom comparison of Varian TrueBeam 4DCBCT and 4DCT. In our study, a comparison of 4DCT and 4DCBCT using motion phantom undergoing regular sinusoidal motion of various amplitude and periods and using the motion patterns from real patients is performed. Motion induced enlargement of a known target volume in three‐dimensional cone beam computed tomography (3DCBCT) is evaluated to estimate the motion amplitude. Motion amplitude was measured using all phases, and only 0% and 50% phases in 4DCT, 4CBCT Advance and 4DCBCT Basic algorithms. The measured volume of a known moving target is compared between 3DCBCT, and the averages of phases from 4DCBCT and 4DCT, and between the maximum intensity projections (MIP) of phases from 4DCBCT and 4DCT. Additionally, the phase‐to‐phase variation in the measured volume is analyzed for both 4DCBCT and 4DCT. Volume comparisons among various imaging methods only considered the 4DCBCT Advance algorithm.

## 2 MATERIALS AND METHODS

### 2.1 Dynamic thorax phantom

The CIRS Model 008A Dynamic Thorax Phantom (Sun Nuclear Corporation, Melbourne, FL) is used to simulate the movement of a lung tumor within the thoracic cavity.^25^ The phantom includes a lung‐equivalent imaging rod with an embedded, 2.5 cm diameter spherical, soft‐tissue equivalent target. The imaging rod can be linearly translated to simulate SI target motion. Using the imaging rod's rotational motion along with the off‐axis location of the target, both anterior‐posterior (AP) and left‐right (LR) motions of the target can be obtained. However, these two motion directions are correlated. Linear and rotational motion of the imaging rod are controlled by the motion actuator box. The manufacturer specified position and time accuracy are given as ± 0.1 mm and ± 0.1 s respectively.^25^ The phantom is equipped with a surrogate platform on which an infrared marker was placed in our study to track motion using respiratory gating for scanner (RGSC, Varian Medical Systems, Palo Alto, CA). The waveform obtained from the RGSC is used to bin the images into respiratory phases.

### 2.2 Regular and irregular waveforms

**FIGURE 1:**
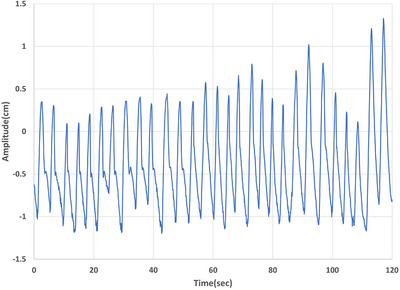
Graph representing one patient's irregular breathing waveform in the SI direction for a duration of 120 seconds.

Regular periodic motion was programmed as a Cos^4^ motion waveform with varying amplitude and period in each direction, while the irregular motions were based on surrogate breathing waveforms of nine patients. A Cos^6^ waveform that has a longer flat valley and faster rise to peak amplitude could have been used but Cos^4^ was adequate to show within‐phase blurring due to fast rise to the peak amplitude. The peak‐to‐peak amplitude of Cos^4^ varied in SI direction between 10–30 mm, in LR and AP directions between 2–5 mm, and the period varied between 3–6 seconds. In this study, the breathing signals of nine patients were used to acquire 4DCT and 4DCBCT images of the motion phantom. These breathing waveforms consist of irregular breathing patterns, as employed by Suh *et al*.^26^ The waveforms came from motion tracking of the tumor via implanted fiducials for patients treated using CyberKnife (Accuray Incorporated, Sunnyvale, CA). The breathing signal data is acquired using a surrogate; however, the waveform is periodically modified to the detected position of fiducials in the stereoscopic 2D X‐ray projection images. This system periodically updates a correlation model between external surrogates and the internal fiducials’ motions, thus estimating the tumor's centroid position by the external surrogate signal. For this study, motion waveform in the SI direction was used (see Figure 1). Since the motion amplitude in the SI direction was very small for some patients, motion amplitudes for all patients were scaled up by a factor of four. Amplitude scaling increases the motion slope but does not affect the spectral content of patient waveforms. The resulting scaled waveform for the first 120‐second segment was extracted and repeated in a loop to move both the surrogate platform, and the imaging rod in the SI direction.

### 2.3 Acquisition of 4DCT

4DCT images were acquired using a Philips Big Bore RT CT (Philips Healthcare, Cleveland, OH) scanner (120 kV, 600 mAs without tube current modulation, 35.5 mGy CTDI_vol_, body bowtie) and reconstructed with 0.68 × 0.68 mm^2^ pixel size and 2 mm slice thickness. We did not use iterative model reconstruction but used standard noise reduction (iDose^4^ level 3, reconstruction filter B). 4DCT reconstructions are inherently retrospective since projections are binned after complete data collection. Since the system acquires 4DCT data using a low‐pitch helical method, we adjusted the helical pitch (0.04, 0.07, 0.09) according to the regular waveform period.^27^ The 4DCT images were binned in 10‐equispaced phases; 0% and 50% represent the highest and the lowest positions, respectively, of the infrared marker on the surrogate platform. The images can also be binned in 10‐equispaced amplitudes from the lowest to the highest position of the infrared marker. For regular breathing motion, only phase binning was used due to the regularity in both the phase and amplitude of the waveform. For irregular motion, both phase and amplitude binning were performed for three patients to study the difference between binning. These three patients’ waveforms had small (< 10 mm), medium (< 20 mm) and large (> 20 mm) motion amplitudes measured by the phase‐binned 4DCT. The smallest pitch (0.04) was used to reduce the binning artifacts caused by any irregularity in breathing period.

### 2.4 Acquisition of 4DCBCT

**FIGURE 2:**
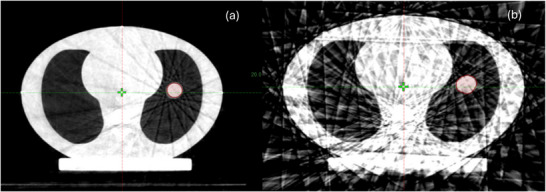
Display of 4DCBCT 0% phase image, reconstructed using (a) Advance algorithm with visibly less artifacts and (b) Basic algorithm, consisting of streak artifacts due to the smaller number of projections per phase [Window: −1000 to 0 HU].

4DCBCT images in 10 phases were acquired on a Varian TrueBeam system (Varian Medical Systems, Palo Alto, CA). 4DCBCT data acquisition used 125 kV, 672 mAs (CTDI_vol_ = 10 mGy compared to 35.5 mGy for 4DCT), 120 second scan time, half‐fan bowtie filter and 84 projections per phase. Additional parameters such as frame rate (7 frames/second) and projection spacing (0.428° per view) were unmodified from default. Please note that bow‐tie filter is present both in calibration air‐scan and phantom scan, its attenuation profile is automatically corrected in projection data processing. An anti‐scatter grid is used to reduce scatter in each projection, and a model‐based scatter correction is applied to each projection before reconstruction.^23^ The phantom setup and motion parameters were the same as the 4DCT imaging, and images were reconstructed using Basic and Advance algorithms for regular waveform. Additionally, 3DCBCT scans were obtained for all regular motions. For irregular waveforms, only the Advance algorithm was used except as noted in section 2.7, since the Basic algorithm resulted in severe artifacts (2 mm slice thickness, phase binning). Figure 2 shows the 0% phase 4DCBCT images which are reconstructed using Advance and Basic reconstruction algorithms.

### 2.5 Analysis of regular motion

The target was delineated in each phase using consistent image thresholding in contouring section of treatment planning system (Eclipse version 15.6, Varian Medical Systems, Palo Alto, CA). A volume of interest was placed around the target within lung and the bi‐model Hounsfield Unit (HU) histogram determined. The threshold was determined consistently for all image sets as the mid‐point between lung and target HU histogram peaks. The centroid coordinates of the target were used to determine the amplitude of motion in all directions. For each regular programmed motion, the coordinate points in all ten phases should ideally lie on a Cos^4^ curve. Using MATLAB's curve fitting tool (Mathworks, Natick, MA. Version 2019) motion amplitude in SI, LR, and AP directions was measured by fitting a Cos^4^ curve to target positions in each phase. The curve fitting uses unconstraint linear least square method for fitting and provides root‐mean‐squared‐error (RMSE) between the measured centroid position in each phase and the Cos^4^ function. The RMSE between the fit and measured centroid coordinates ranged from 0.5 mm (10 mm SI motion) to 1.7 mm (30 mm SI motion) and 95% confidence intervals (CI) for estimated motion amplitudes ranged from 9.9–10.1 mm (10 mm SI motion) to 27–33 mm (30 mm SI motion). The RMSE ranged from 0.1 mm (4 mm AP and LR motion) to 0.5 mm (10 mm AP and LR motion) and 95% CI ranged from 3.8–4.2 mm (4 mm AP and LR motion) to 9.9–10.1 mm (10 mm AP and LR motion). Measured centroid coordinates along the upslope and downslope of motion curve mainly contributed to the RMSE; thus, the larger motion amplitudes with larger up/down slopes have larger RMSE and wider 95% CI. Additionally, partial volume effect may also contribute to RMSE for these cases. Differences between the programmed and measured motion amplitudes were calculated for all scanning methods and for all programmed motions.

The motion amplitudes were also measured as the differences between target centroid coordinates in 0% and 50% phases, and the difference between the programmed and measured amplitudes was calculated. The mean and standard deviation of the differences between the measured and programmed motions were computed for all three directional axes (SI, AP and LR) and all imaging techniques.

The motion amplitude was also measured using 3DCBCT; the current image guidance method in lung SBRT. The profiles through the target's center in both the coronal and sagittal planes of 3DCBCT images represent the motion blurred size of the target. The blurring in 3DCBCT, in general, should scale with target velocity and exposure time. The horizontal profiles in the coronal and sagittal planes were used to measure the LR and AP motion amplitudes respectively. The vertical profiles in both coronal and sagittal planes were used to measure the SI motion amplitude. The motion amplitudes were estimated by subtracting the known target size from the Full Width at 5% of Maxima (FW5%M) in each profile; 5% was determined as the lowest downward value in the profiles above the CT number variation caused by motion artifacts. Previous investigations^20^, ^28^ used manual measuring tools along with display window‐level adjustment to determine the target elongation in 3DCBCT for SI motion only. We chose to standardize this measurement using the FW5%M to reduce measurement variation which may occur in placing manual tool in the image. An example LR with 5% threshold in a coronal CBCT image is shown in supplemental Fig. S1. However, the manual method was used to verify our method in the SI direction. The measurement error of motion amplitude using the manual method was consistent with the standardized method; there was no significant difference in error between two methods (*p* = 0.38). Due to significant artifacts, we did not find a consistent method of manually measuring motion amplitude in AP and LR directions. The mean and standard deviation of the difference between the programmed and measured motion amplitude were determined.

### 2.6 Target volume analysis

Target volumes were measured in the MIP and AIP through phase images of both 4DCT and 4DCBCT within the treatment planning system. The 3DCBCT target volume was also compared with the AIP images. The target volume in each phase for 14 and 30 mm SI motions (averaged over three cases of breathing period: 3, 4, 6 second) was determined to see if the target volumes vary with phase. For these 2 cases of SI motion, any effect of breathing period on measured target volume was also determined by averaging the target volume over all phases for each breathing period.

### 2.7 Patient waveform analysis

In total, nine patient‐specific irregular waveforms were analyzed. For each of the nine patients, 4DCT was performed, and the data was reconstructed using phase binning. Only the Advance reconstruction algorithm was used in 4DCBCT imaging as the Basic algorithm typically produced streak artifacts. The irregularity of the breathing waveforms exacerbated these artifacts. Additionally, amplitude binning was performed for three arbitrarily selected patients to compare the binning method for irregular waveforms. For the same patients, Basic 4DCBCT images were also reconstructed.

The target in each image dataset was delineated using the same threshold‐based contouring as used for regular waveforms, but due to streaking and motion artifacts, manual correction of the target contour was required. To assess the intra‐observer variability, the auto‐contouring and manual correction for a 0% phase image was repeated from scratch on eight separate days by a single user. Manual corrections began with auto‐contours each day to reduce memory bias of a single user. The standard deviations and range (around the mean) in eight separate measurements of target coordinates in a 0% phase image were determined to assess the intra‐observer variability in manual contour corrections. Standard deviations (ranges) were 0.4 mm (−0.5 to 0.4 mm), 0.2 mm (−0.3 to 0.2 mm) and 0.2 mm (−0.3 to 0.3 mm) in LR, AP and SI directions respectively. The difference in target centroids between 0% and 50% phases provided the measured amplitude in the SI motion of the patient waveforms. The measured amplitudes were compared with the programmed amplitude. The programmed amplitude was taken as the average amplitude from 120 seconds of patient waveform after correcting for phantom limitations.

## 3 RESULTS

### 3.1 Regular motion

**FIGURE 3:**
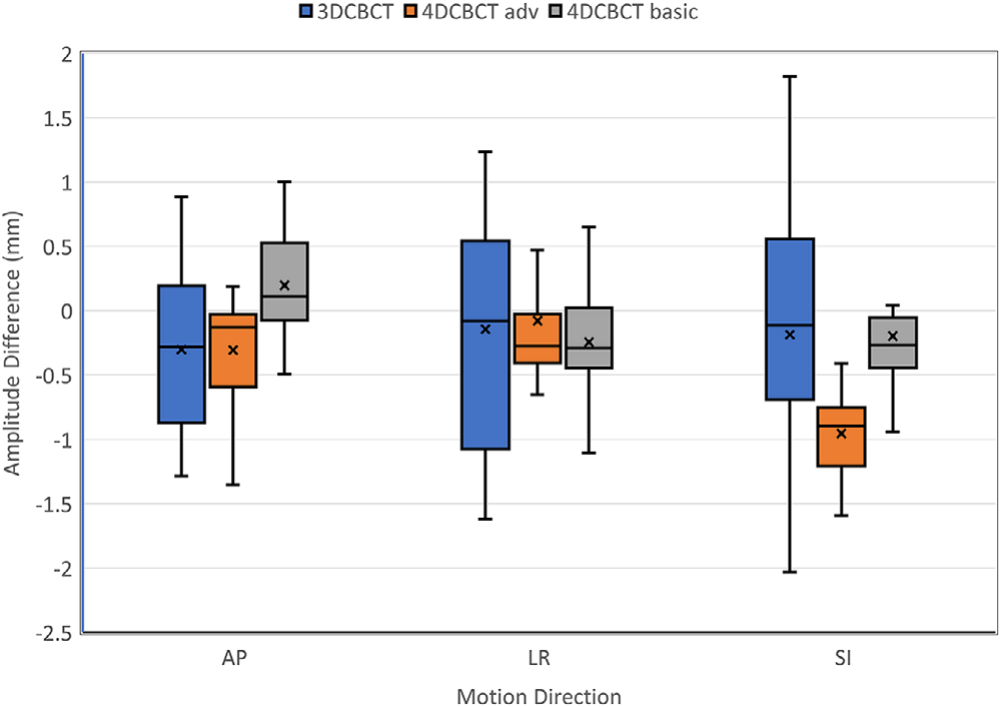
The differences between the measured and programmed motion amplitudes using 3DCBCT, 4DCBCT (Advance) and 4DCBCT (Basic) imaging methods as box‐whisker plots with outliers excluded in 4DCBCT due to artifacts (Basic:1.6 mm AP, Advance: 2.1 mm LR). The lines and crosses within the box represent the median and average in each case.

Figure 3 shows the differences between the measured and programmed amplitude in SI, AP and LR direction from 3DCBCT, 4DCBCT (Advance) and 4DCBCT (Basic), excluding the outliers in 4DCBCT due to artifacts (Basic:1.6 mm AP, Advance: 2.1 mm LR). The inter‐quartile range and min‐max range is an indicator of measurement confidence. Table 1 provides the median error and its inter‐quartile range for each case presented in Figure 3. These metrics, in general, are larger in the 3DCBCT measurements compared to either of the 4DCBCT measurements. This is because the motion is indirectly estimated in 3DCBCT as it does not explicitly resolve the motion trajectory. Table 2 presents standard deviation and 95% CI of the differences between the measured (curve fit to all phases) and programmed motions. The median and 95% CI are the smallest in 4DCT due to the better in‐plane resolution and image contrast in images compared to 4DCBCT images.^29^ The two reconstruction methods in 4DCBCT provide comparable results for regular motion. The larger 95% CI in 4DCBCT was attributed to fewer projections collected per phase, leading to under‐sampling and streaking artifacts.^30^ Median SI motions measured by 4DCT and the Basic 4DCBCT are closer to the programmed one, while the Advance 4DCBCT reconstruction measures smaller than the programmed motion, nearly 1 mm (*p* < 0.05 using Wilcoxon Signed‐Rank Test). In LR and AP directions, the differences between three imaging methods were not statistically significant (*p* > 0.05 using Wilcoxon Signed‐Rank Test). Advance 4D method reconstructs a *prior* image using all the projections. The *prior* image should ideally just be the motion blurred image of moving objects, but it contains streaking artifacts especially emanating from the moving high contrast objects such as ribs, moving soft‐tissue tumors in the middle of lung, and moving fiducials in lung or liver. This *prior* image is reprojected at the acquisition angles and subtracted from the acquired projections to create projections of the moving‐only objects. Phase images of the moving‐only projections are reconstructed and added to the *prior* image to create final phase images. In principle, this method is supposed to reduce view aliasing streaking artifacts for stationary objects and encode motion in the final phase images. However, due to the streaking artifacts in the *prior* image, final phases may contain ghosting.^23^ Although the Advance 4DCBCT method is supposed to reduce this ghosting by reducing the streaking artifacts in the *prior* image, residual ghosting remains which interferes with the auto‐contouring of the target and creates systematic bias in the measured motion amplitude (e.g. mean SI motion underestimation ≈ 1 mm)^23^ even if the images appear better than Basic 4DCBCT method.

**TABLE 1:** Median and inter‐quartile range of amplitude differences in regular motion.

	Median, (Inter‐quartile Range) mm		
Imaging method	LR	AP	SI
4D CBCT advance	−0.3 (0.4)	−0.1 (0.6)	−**0.9 (0.5)**
4D CBCT basic	−0.3 (0.5)	0.1 (0.6)	−**0.3 (0.4)**
3DCBCT	−0.1 (1.6)	−0.3 (1.1)	**0.0 (1.2)**

**TABLE 2:** Amplitude differences in regular motion from all phases.

Imaging method	Standard deviation, (95% Lower, 95% Upper) mm		
	LR	AP	SI
4D CT	0.1, (−0.3, 0.2)	0.1, (−0.5, 1.1)	**0.2, (**−**0.8, 0.1)**
4D CBCT advance	0.6, (−1.4, 1.2)	0.4, (−1.2, 0.6)	**0.3, (**−**1.5,** −**0.4)**
4D CBCT basic	0.4, (−1.1, 0.6)	0.6, (−0.9, 1.3)	**0.5, (**−**1.1, 0.7)**
3DCBCT	0.9, (−1.9, 1.6)	0.7, (−1.6, 1.0)	**0.9, (**−**1.7, 1.6)**

Table 2 also provides the median and 95% CI of the differences between the measured and programmed motion from 3DCBCT images in regular motion. The 95% CI are in general larger than the analyses that use all phases of 4D imaging methods. The increased 95% CI in 3DCBCT measurements are consistent with the larger inter‐quartile range for 3DCBCT in Figure 3.

### 3.2 Motion analysis using coordinates in 0% and 50% phases

The motion amplitude was also measured using only the 0% and 50% phases for regular motion. The 95% CI of differences between the measured and programmed motions was in general larger than that obtained using the curve fit to all phases (Table 3). The 95% CI of the difference in SI direction is much larger in 4DCBCT measurements when only 0% and 50% phases are used for motion amplitude measurements. For example, the 95% CI in SI direction is increased from 0.9 mm to 2.1 mm in 4DCT, from 1.1 mm to 6.6 mm in 4DCBCT Advance and from 1.8 mm to 6.3 in 4DCBCT Basic. The target centroid measurements have errors due to auto‐segmentation, pixel resolution and slice thickness. These errors are enlarged in subtracting the target centroids between 0% and 50% phases, while the effect of these errors is minimized in least‐squared regression to Cos^4^ curve.

**TABLE 3:** Amplitude differences in regular motion from 0% and 50% phases.

Imaging Method	Mean, (95% Lower, 95% Upper) mm		
	LR	AP	SI
4D CT	0, (−0.3, 0.2)	0.3, (−0.5, 1.1)	0.1, (−0.9,1.2)
4D CBCT advance	−0.1, (−1.9,1.7)	0, (−1.4, 1.4)	0, (−3.3, 3.3)
4DCBCT basic	−0.3, (−1.2, 0.7)	−0.3, (−1.1, 0.6)	0.2, (−2.9, 3.4)

### 3.3 Volume analysis

**FIGURE 4:**
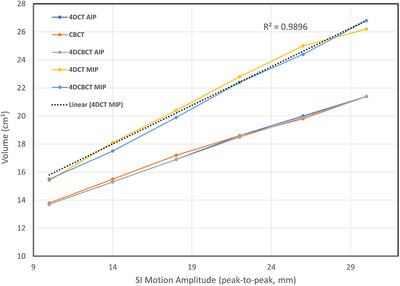
Target volume (cm^3^) in CBCT, CT AIP, CBCT AIP, CT MIP and CBCT MIP images as a function of peak‐to‐peak SI motion amplitude (only for the largest motion in LR and AP directions). The points in AIP images lie on top of each other. The worst case linear fit (CT MIP) is also shown. Volume increase per mm of motion is 0.39 cm^3^ (4DCT AIP), 0.38 cm^3^ (4DCBCT AIP), 0.37 cm^3^ (3DCBCT), 0.57 cm^3^ (4DCBCT MIP), and 0.55 cm^3^ (4DCT MIP).

In regular motion, the target volume measured in 3DCBCT, and in the AIP images of 4DCBCT (Advance) and 4DCT are nearly the same. Mean and standard deviation of the volume difference between the AIP image of 4DCT and 3DCBCT is ‐0.06 ± 0.1 cm^3^, and between the AIP images of 4DCBCT and 4DCT is 0.04 ± 0.07 cm^3^. These results are expected since 3DCBCT reconstruction using all projections is spatially equivalent to average of all individual phase images; however, the 3DCBCT cannot measure the target volume in individual phases. Similarly, the measured target volumes in the MIP images of 4DCT and 4DCBCT are nearly the same. The mean and standard deviation of the differences in volume between two MIP images is 0.18 ± 0.5 cm^3^. As expected, the target volume in the MIP images is significantly larger than the AIP images of 4DCT and 4DCBCT (Figure 4), and the volumes are linearly increasing with the motion amplitude (worst case R^2^ = 0.9896, for 4DCT MIP).

**FIGURE 5:**
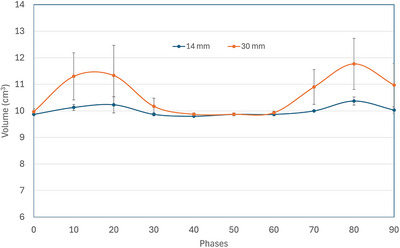
Graph representing variation in target volume (cm^3^) measurement in all phases for 14 and 30 mm SI motion in 4DCT.

**FIGURE 6:**
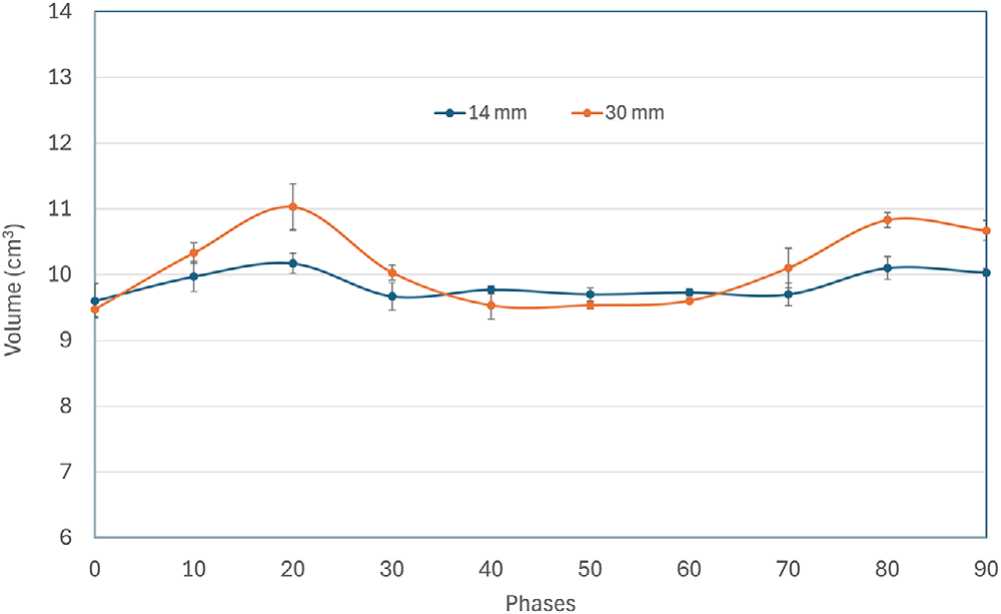
Graph representing variation in target volume (cm^3^) measurement in all phases for 14 and 30 mm SI motion in 4DCBCT Advance

The measured target volume in each phase, averaged over three cases of motion period, for the SI motion amplitudes of 14 and 30 mm is presented in Figure 5 and 6, respectively, for 4DCT and 4DCBCT Advance. In the absence of within‐phase motion blurring, the target volume in each phase should ideally be independent of motion amplitude and phase. As can be seen in Figures 5 and 6, this is only true for 0% phase, and the phases around 50%. These phases represent the smallest slope in the motion waveform and have the minimal within‐phase motion blurring. The motion waveform slope is the largest around 20% and around 80% phases which show the largest difference in target volume between 14 and 30 mm motion amplitudes. The general behavior of target volume vs. phase curves is largely similar between 4DCT and 4DCBCT.

As the measured target volume depends on the motion slope, it may depend on the breathing period for a given programmed amplitude. We measured the average target volume of all phases, for each motion period separately, of both 4DCT and 4DCBCT (Advance) for 14 and 30 mm motions in SI direction. The average target volume's dependence on breathing period was noticed only for 30 mm SI in 4DCT (11.1 ± 1.2 cm^3^ for 3 sec and 10.2 ± 0.4 cm^3^ for 6 sec period); this dependence came primarily from phases where the target velocity is larger. For the 0% and 50% phases, this decrease was negligible. For 14 mm SI motion in 4DCT, and for both motions in 4DCBCT, the breathing period dependence of the average target volume was not significant. The target volume for 14 (30) mm motion, averaged over all phases and periods, were 10.0 ± 0.2 cm^3^ (10.6 ± 0.9 cm^3^) and 9.8 ± 0.2 cm^3^ (10.1 ± 0.6 cm^3^) for 4DCT and 4DCBCT respectively. The changes in the averaged target volume were statistically significant with respect to change in motion amplitude and with respect to imaging modality, these changes are not expected to be clinically significant.

### 3.4 Irregular patient waveforms

Target motion amplitudes for nine patients’ irregular breathing waveforms were measured using centroid coordinates of phantom target in 0% phase and 50% phase images of 4DCT and 4DCBCT Advance images. Amplitude measured by 4DCT is closer to the programmed motion than 4DCBCT for some waveforms, and vice versa for the others Supplemental Table (S1). Patient 5 shows the maximum difference for 4DCT between the measured and the programmed motion amplitudes while Patient 2 has the largest difference for 4DCBCT. The sources of error in 4DCT include short total scan time (≈ 69 seconds) compared to 120 seconds period of repetition of motion waveforms in the loop, and the binning artifacts due to irregularity in amplitude and period of breathing waveforms. 4DCBCT Advance also suffers from binning artifacts due to irregular breathing in addition to double target image effects caused by the Advance 4DCBCT reconstruction method. The programmed motion amplitude itself is an estimate from the otherwise irregular motion. Table S1 shows that there is not a clear‐cut advantage of 4DCT to 4DCBCT, and vice versa, a paired two‐tail t‐test supported no difference (Pearson Correlation Coefficient = −0.25 with *p* = 0.82). There could be differences between 4DCT and 4DCBCT measured amplitudes up to 6 mm (Patient 9).

4DCBCT image data sets for three irregular patient breathing waveforms were acquired and reconstructed using both Basic and Advance image reconstruction algorithms. Amplitude measured by 4DCBCT Advance method is slightly closer to the programmed amplitude than 4DCBCT Basic method for the patient breathing waveform shown in Table 4. Images of 4DCBCT Basic contain stronger streaking artifacts as compared to 4DCBCT Advance that get worse due to the irregularity in motion.

**TABLE 4:** Basic and advance reconstruction in irregular motion.

Patient	Phantom (mm)	4D CBCT basic (mm)	4D CBCT (advance) (mm)
2	29.94	21.9	25
5	10.8	7.4	7.6
9	13.99	8.1	9.1

Amplitude binning for 4DCT and 4DCBCT images for 3 patient waveforms were also performed (Table 5). Comparison of phase and amplitude binning for these patients shows that, in case of 4DCT, the phase binning gives better results compared to the amplitude binning. Especially for patient 5, the amplitude binning for 4DCT represents the worst results. In amplitude binning, the local maxima (peaks) and minima (valleys) points on the waveform are selected, and average peak and valley are determined excluding the outlier points.^12^ The algorithm will target the 0% and 50% bins to the average peak and valley points respectively. Points that exceed these boundaries are not utilized when reconstructing amplitude binned images, thus outliers are thrown away. Due to this, some motion information is lost which may result in less accurate measurement of the motion although visual binning artifacts are significantly reduced in the amplitude binned images. The phase binning, which includes all amplitudes, may be better in some patients while amplitude binning might misrepresent motion because outliers are thrown away. The breathing waveform of patient 5 shows large irregularities in amplitude especially in time range from 0 to 50 seconds. As 4DCT takes significantly less than 120 seconds for data acquisition, it is not known when the 4DCT is acquired within the repeating 120‐second motion waveform. This is possibly the reason that patient 5 has the largest difference between the two, phase binning measurements, i.e. 6.9 mm in Table S1 and 8.6 mm in Table 5. Comparing 4DCBCT phase and amplitude binning methods shows almost similar results. In case of patient 5, 4DCBCT gave better result than 4DCT as 4DCBCT takes 2 minutes for acquisition of images therefore included entire variation of motion the waveform.

**TABLE 5:** Amplitude and phase binning in irregular motion.

Patient	Phantom (mm)	4D CT amplitude binning (mm)	4D CT phase binning (mm)	4D CBCT amplitude binning (mm)	4D CBCT phase binning (mm)
2	29.94	24.8	**26.9**	23.4	**24.1**
5	10.8	3.5	**8.6**	8	**7.3**
9	13.99	10.5	**14.7**	10.2	**9.5**

## 4 DISCUSSION AND CONCLUSION

3DCBCT is the most used image‐guidance technique for lung SBRT; however, it does not explicitly resolve tumor motion due to breathing. In contrast, 4DCBCT captures tumor motion immediately prior to treatment. Since 4DCT (on CT simulators) and 4DCBCT (on treatment delivery systems) are implemented differently, it is essential to validate the consistency of motion measurements between these systems. Several studies have evaluated the XVI 4DCBCT system's (Elekta, Stockholm, Sweden) in terms image quality and motion trajectory accuracy,^14^ on‐line localization accuracy,^20^ target motion amplitude measurement accuracy^13^ and target volume measurement accuracy.^30^ Similar evaluations of the Varian's 4DCBCT system (OBI, Varian Medical Systems, Palo Alto, CA) are needed. In this study, we evaluated the OBI's 4DCBCT in terms of motion and volume measurement accuracy. Both regular cosine and irregular patient waveforms were used to move a known target.

Motion amplitude measured using all phases provided the smallest 95% CI of the difference. However, as explained in section 3.1, the Advance 4DCBCT gave a larger mean difference in SI direction. Using only the 0% and 50% phases had much larger 95% CI in SI direction compared to curve fitting method. In our study, the measured motion amplitude in 4DCT was closer to the programmed amplitude compared to any of the 4DCBCT methods. This contrasts with Baley *et al* ^13^ where the 4DCBCT measurements were closer to the ground truth, possibly because of larger slice thickness (2.5 mm) used and appearance of motion artifacts in their 4DCT. In both studies, the differences between the 4DCT and 4DCBCT results based on curve fitting method are small. In our study, the maximum difference between programmed and measured motion amplitudes was 1.1 mm for 4DCT and 1.6 mm (Basic) and 2.1 mm (Advance) for 4DCBCT. Overall, 4DCBCT yielded motion measurements within 2 mm of 4DCT. In Baley *et al*, (Table S3), the maximum difference between the measured and programmed motions was 1.1 mm for 4DCT (2.5 mm slice thickness) and 1.4 mm for 4DCBCT (1 mm slice thickness). Our maximum differences for 4DCBCT are larger possibly due to the 2 mm slice thickness used. For regular motion, the 3DCBCT method gave a larger 95% CI of difference compared to 4DCBCT. Thus, when motion is expected to change between planning CT and localization imaging, it may be beneficial to use 4DCBCT imaging due to increased confidence in motion measurements and explicit motion resolution in phases.^20^

For irregular motion, the difference between 4DCT and 4DCBCT measurements are less than 6 mm in our study which is comparable to Baley *et al*,^13^ however, these differences in general are larger in our study possibly due to the larger slice thickness used in 4DCBCT. We also found that the Advance reconstruction algorithm in Varian's 4DCBCT, although giving a larger mean difference in SI direction for regular motion, was closer to 4DCT measurements for 3 patients than the Basic method. Thus, the artifact reduction by Advance algorithm may have a benefit in irregular motion. For irregular motion, amplitude binning provided the worse result in 4DCT while the binning method was not so important in 4DCBCT Advance. Particularly, 4DCT amplitude binning method throws away outliers in putting binning levels, and its use should be carefully considered based on the irregularity of waveform.

It should be noted that irregular waveforms were used to highlight differences in the implementation of 4DCT and 4DCBCT. Breathing cycle to cycle differences in irregular waveform manifest as slice‐to‐slice differences in 4DCT, e.g. not every slice is at the same amplitude level in 50% phase set although individual slices may not show artifacts. In 4DCBCT, every projection in 50% phase bin may be at a different amplitude of motion resulting in artifacts in the reconstructed image. In addition, 4DCT total scan time is significantly smaller than 120 seconds, as such, 4DCBCT sees the entire 120 second irregularity in waveform which potentially makes 4DCBCT measurement closer to the average amplitude. Therefore, neither the differences between measured and programmed amplitudes for each imaging method, nor the differences between two methods are indicative of the localization errors of image guidance. This is because image‐to‐image registration of AIP or phase composite displayed images is used to localize the tumor in image guidance in practice, and the motion amplitude measurements made using only 0% and 50% phases are likely more sensitive to implementation differences. Thus, patient studies are needed to determine the advantage of 4DCBCT in localization using image guidance for the Varian's implementation.

Target volume measurements were made in our CBCT, the AIP images of 4DCT and 4DCBCT, and the MIP images of 4DCT and 4DCBCT, all for regular waveforms. Target volumes in AIP and MIP were consistent between 4DCT and 4DCBCT. Tzu‐Cheng Lee *et al* ^30^ compared 4DCT and XVI 4DCBCT in terms of lesion volume accuracy for 10 sinusoidal motion waveforms. The authors observed that the target volumes, averaged over all phases and breathing periods, were significantly larger in 4DCBCT than 4DCT, e.g. 8.2 ± 0.2 cm^3^ in 4DCBCT vs. 5.5 ± 1.4 cm^3^ in 4DCT for 30 mm motion. In our results, 4DCBCT target volume was slightly less, not expected to be clinically significant, than 4DCT volume. Their results showed a greater volume increase with an increasing breathing period in 4DCT than ours, which showed a noticeable decrease in volume only in 4DCT for 30 mm SI motion.

Moreover, contrary to Tzu‐Cheng Lee *et al*,^30^ the target volume change as a function of breathing phase showed very similar behavior in our measurements between 4DCT and 4DCBCT, i.e. volume in 20% and 80% phases increased due to within‐phase blurring caused by larger target velocity. The target volume showed a huge reduction in these phases for 4DCT and a small noticeable reduction in 4DCBCT in Tzu‐Cheng Lee *et al* study. The differences between cine based and low‐pitch helical 4DCT may account for this difference in behavior for 4DCT. Our results are consistent with the larger motion blurring ratio (MBR) in mid‐inhale phase reported by Soyoung Lee *et al* ^14^ although, contrary to,^14^ the “MBR” did not depend on the breathing period in 4DCBCT. Kejda *et al* ^31^ compared the XVI and OBI in terms of ITV between 4DCT and 4DCBCT. Linear volume increase with motion amplitude observed in this study is also consistent with our study.

This study is limited regarding the use of low‐pitch helical 4DCT for irregular breathing waveform since intelligent 4DCT (i4DCT) systems are now available.^8^, ^9^, ^10^ This cine‐based 4DCT data collection analyzes the patient breathing waveform to adapt the scan duration at each couch position to acquire adequate data. This method has the potential to reduce image artifacts such as missing or incorrectly binned moving targets and potentially be more accurate than low‐pitch helical 4DCT in irregular motion. Future work will consider comparing i4DCT with the current version of 4DCBCT.

The major aim of our study was to determine if image guidance in lung SBRT using 4DCBCT is potentially more beneficial than the current 3DCBCT approach. Our results show that 3DCBCT provides target volume enlargement like AIP image from 4DCBCT or 4DCT. However, the indirect estimate of target motion has poor confidence compared to 4DCBCT. These results indirectly indicate that CBCT may be less useful if the motion is expected to change between planning 4DCT which was also the conclusion from,^20^ a study based on the XVI system. Our results also show that 4DCBCT is very accurate in determining motion amplitude and measures the target volume consistent with 4DCT both in average and MIP. Moreover, the target volume enlargement due to within‐phase blurring is similar between 4DCT and 4DCBCT which makes 4DCBCT useful for localization when mid‐inhale phase is used for treatment planning. Due to implementation differences, motion amplitudes can be different between 4DCT and 4DCBCT for some cases of irregular breathing; more work is needed to understand these differences. At the same time, 4DCBCT requires longer acquisition time (60 seconds more) and delivers higher imaging dose (≈ 10 mGy) compared to 3DCBCT (≈ 3–5 mGy). 4DCBCT demonstrates accuracy within ∼ 2 mm of 4DCT for regular motion and comparable performance for irregular motion. While promising for SBRT image guidance, higher imaging dose and longer acquisition time limit its routine use since integration into routine image guidance workflows also depends on dose tolerance, treatment‐time constraints, and clinic‐specific protocols.

## AUTHOR CONTRIBUTIONS

Bhumika Handa: This work is direct result of Bhumika's MSc. thesis. This author collected all the data and analyzed the data. This author also produced the first draft of the manuscript. Satyapal Rathee: This author conceived the project and provided supervision to the first author during graduate work. This author also reviewed the data collection and analysis and revised the manuscript several times to provide feedback to the first author.

## CONFLICT OF INTEREST STATEMENT

The authors have no relevant conflicts of interest to disclose.

## Supporting information



Supporting information

Supporting information

Supporting information

Supporting information

## Data Availability

All usable data is provided within the manuscript.
