# Supplementary material for: Comparative evaluation of 4DCT and 4DCBCT for motion and volume measurement accuracy in a dynamic phantom
Source: J Appl Clin Med Phys. 2026 Feb 24;27(3):e70489. doi: 10.1002/acm2.70489 (PMC12931249; doi:10.1002/acm2.70489)
Supplement: Supplementary file 1 — Supporting information [file ACM2-27-e70489-s001.docx]

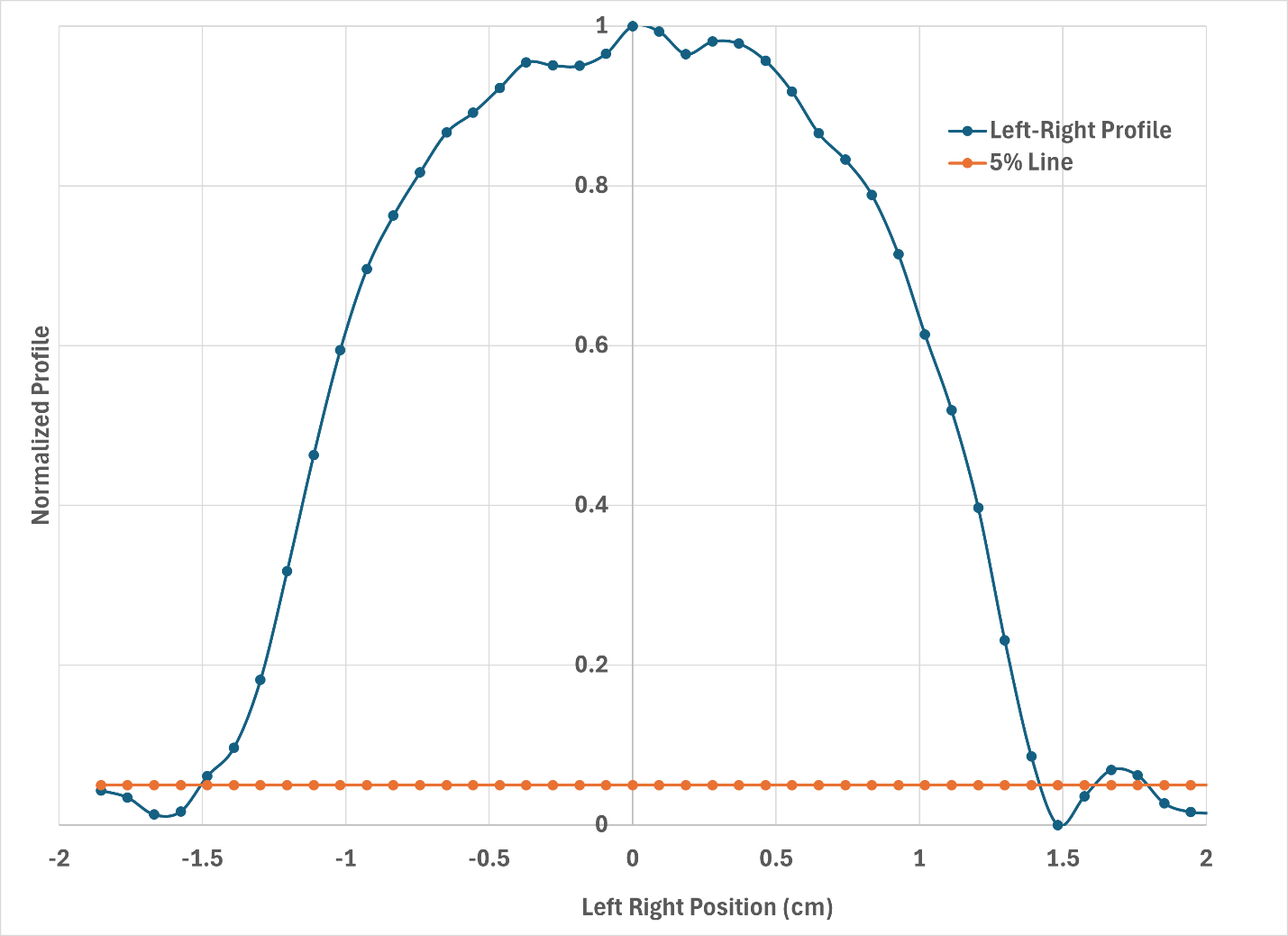


Figure S1: An example horizontal profile through phantom target in a coronal CBCT image. The profile is normalized to 1.0 at the maximum value. The red line represents the 5% threshold to determine the full width at 5% of maximum (FW5%M). FW5%M is used to determine the blurred size of the target and an estimate of the motion amplitude.
