# Supplementary material for: Comparative evaluation of 4DCT and 4DCBCT for motion and volume measurement accuracy in a dynamic phantom
Source: J Appl Clin Med Phys. 2026 Feb 24;27(3):e70489. doi: 10.1002/acm2.70489 (PMC12931249; doi:10.1002/acm2.70489)
Supplement: Supplementary file 2 — Supporting information [file ACM2-27-e70489-s002.docx]

| **Patient** | **Phantom(mm)** | **4D CT (mm)** | **4D CBCT (Advance) (mm)** | **Diff between 4DCT and phantom (mm)** | **Diff between phantom and 4DCBCT (mm)** |
| --- | --- | --- | --- | --- | --- |
| 1 | 21.37 | 18.9 | 23.5 | 2.47 | -2.13 |
| 2 | 29.94 | 26.8 | 25 | 3.14 | **4.94** |
| 3 | 8.9 | 9.7 | 7.4 | -0.8 | 1.5 |
| 4 | 36.72 | 35.9 | 35.7 | 0.82 | 1.02 |
| 5 | 10.8 | 6.9 | 7.6 | **3.9** | 3.2 |
| 6 | 13.08 | 9.7 | 12.7 | 3.38 | 0.38 |
| 7 | 2.52 | 1.6 | 1.3 | 0.92 | 1.22 |
| 8 | 13.49 | 10.4 | 15.2 | 3.09 | -1.71 |
| 9 | 13.99 | 15.1 | 9.1 | -1.11 | 4.89 |

Table S1: Amplitude of phantom target motion for irregular patient breathing waveforms of 9 patients as measured using 4DCT and 4DBCT Advance. The phantom motion magnitude is an average over 120-second waveform and corrected for phantom limitations. The difference in amplitudes (mm) between programmed and measured values are also given for 4DCT and 4DCBCT images.
