# Supplementary material for: Comparative evaluation of 4DCT and 4DCBCT for motion and volume measurement accuracy in a dynamic phantom
Source: J Appl Clin Med Phys. 2026 Feb 24;27(3):e70489. doi: 10.1002/acm2.70489 (PMC12931249; doi:10.1002/acm2.70489)
Supplement: Supplementary file 3 — Supporting information [file ACM2-27-e70489-s004.docx]

| Imaging Method | 95% inter-quartile width (mm) | | |
| --- | --- | --- | --- |
|  | LR | AP | SI |
| 4D CBCT Advance | 0.4 | 1.4 | **1.0** |
| 4D CBCT Basic | 0.5 | 1.7 | **1.7** |
| 3DCBCT | 1.6 | 2.1 | **3.0** |

Table S2: 95% inter-quartile width of the differences between the measured and the programmed motion amplitude for 4DCBCT Advance, 4DCBCT Basic, and 3DCBCT data presented in Fig. 3.

.
