# Supplementary material for: Comparative evaluation of 4DCT and 4DCBCT for motion and volume measurement accuracy in a dynamic phantom
Source: J Appl Clin Med Phys. 2026 Feb 24;27(3):e70489. doi: 10.1002/acm2.70489 (PMC12931249; doi:10.1002/acm2.70489)
Supplement: Supplementary file 4 — Supporting information [file ACM2-27-e70489-s003.docx]

| Imaging System and Motion | This Work (mm) | Baley *et al* (Ref. 13) (mm) |
| --- | --- | --- |
| Regular Motion 4DCT | 1.1 | 1.1 |
| Regular Motion 4DCBCT | 2.1 | 1.4 |
| Irregular Motion, between 4DCT and 4DCBCT | 6.0 | 6.2 |

Table S3: A comparison of the maximum difference between the measured and programmed amplitude for regular motion, and maximum difference between 4DCT and 4DCBCT for irregular motion between this work and Baley *et al* (Ref. 13)
